# Effects of agricultural landscape structure and canola coverage on biochemical and physiological traits of the ground beetle *Poecilus cupreus*

**DOI:** 10.1007/s10646-023-02701-3

**Published:** 2023-09-27

**Authors:** Grzegorz Sowa, Agnieszka J. Bednarska, Ryszard Laskowski

**Affiliations:** 1 Institute of Environmental Sciences, Jagiellonian University, Gronostajowa 7, 30-387 Kraków, Poland; 2 Institute of Nature Conservation, Polish Academy of Sciences, A. Mickiewicza 33, 31-120 Kraków, Poland

**Keywords:** Agroecosystem, Carabidae, Survival, Respiration, Acetylcholinesterase, Starvation

## Abstract

The intensifications in the agricultural landscape and the application of pesticides can cause adverse effects on the fitness of organisms in that landscape. Here, we investigated whether habitats with different agricultural pressures influenced acetylcholinesterase (AChE) activity – a biomarker for exposure to pesticides, respiration rate, and resistance to starvation in the ground beetle *Poecilus cupreus*. Two differently structured landscapes were selected for the study, one dominated by small (S) and another by large (L) fields. Within each landscape three habitat types were selected: in the S landscape, these were habitats with medium (M), small (S) and no canola (meadow, 0) coverage (i.e., SM, SS, S0), and in the L landscape habitats with large (L), medium (M) and no canola (meadow, 0) coverage (i.e., LL, LM, L0), representing different levels of agricultural pressure. The activity of AChE was the highest in beetles from canola-free habitats (S0 and L0), being significantly higher than in beetles from the SM and SS habitats. The mean respiration rate corrected for body mass was also the highest in S0 and L0 beetles, with significant differences between populations from L0 vs. SS and from S0 vs. SS. Only beetles from S0, SS, L0, and LM were numerous enough to assess the resistance to starvation. Individuals from the LM habitat showed better survival compared to the canola-free habitat in the same landscape (L0), whereas in S landscape the SS beetles survived worse than those from S0, suggesting that characteristics of L landscape may lead to developing mechanisms of starvation resistance of *P. cupreus* in response to agricultural pressure.

## Introduction

The intensification of agriculture at the landscape level is blamed for the observed decrease in biomass, abundance, and species diversity of arthropods (Seibold et al. 2019). The intensification of agriculture is associated not only with increasing pesticide use but also leads to a significant transformation of the landscape structure: small fields separated by boundary strips, hedgerows, and other refuges for non-target arthropods are replaced with large-scale monocultures and semi-natural landscape elements are disappearing. Such a simplified landscape dominated by intensively cultivated monocultures may significantly affect the biodiversity, abundance, and population dynamics of non-target arthropods even without pesticide use.

Living in an intensively managed agricultural landscape can result in the development of mechanisms to survive such adverse conditions (Haridas and Tenhumberg 2018). The so-called “general theory of stress” suggests that even a slight adverse change in the environment, which causes the stress state in an organism, is associated with shifts in energy allocation, forcing the allocation of resources to processes counteracting the effects of a stressor (Sibly and Calow 1989). The decrease in available energy reserves in the form of carbohydrates, lipids and proteins can be used as a biomarker of contaminant-inducted stress (Saleem et al. 1998, Even et al. 2012). In case of exposure to toxic chemicals (pesticides), one may, thus, expect that as more energy is allocated to detoxification processes, less energy is available for other maintenance costs and production. In an organism, this can be reflected in a lower gain of body mass, decline in fertility, shorter life span, or reduced resistance to other stressful environmental factors (Stone et al. 2001). In the case of prolonged chronic exposure, such changes may result in specific adaptations to the stressing factor. When it comes to exposure to plant protection products, this is manifested as increased resistance to the most often used insecticides (Jacomb et al. 2016).

According to our previous study (Sowa et al. 2022) on the ground beetle *Poecilus cupreus* (Linnaeus, 1758), resistance in some populations strongly depends on large-scale landscape characteristics. Individuals that inhabited areas with medium and high canola coverage in the landscape dominated by large fields were less sensitive to insecticide exposure than those from a landscape with a more complex structure. The latter one, composed mostly of small fields, with high spatio-temporal diversity of crops and abundant small, linear non-crop elements, may provide shelter that allows beetles to survive without developing any, presumably costly, resistance mechanisms.

The ability of an insect to adapt to the environment contaminated with insecticides depends mainly on effective mechanisms of detoxification of specific chemical compounds (Jokanović 2001). The insecticide-induced production of detoxification enzymes (Shou-Min 2012) is, thus, evidence of resistance to these toxicants. Any stress response, including enzyme production, has the effect of redirecting available energy to protective systems. Due to existing physiological trade-offs, implementing such protective mechanisms may allow keeping the survival rate at the expense of body growth or reproduction (Mallqui et al. 2014). One of the known defence mechanisms against insecticide toxicity involves increased production of acetylcholinesterase (AChE) and/or modifications in the structure of AChE – the key component in the synaptic signal transmission (Kreissl and Bicker 1989). The increased production of AChE or detoxifying enzymes should affect the overall energetic budget of an organism and, in the end, its fitness by increasing the maintenance costs. At the organism level, these should be reflected as an increased respiration rate, making it a potentially useful measure of overall changes in the metabolic rate (Plata-Rueda et al. 2020). The increased tolerance to a stressor may, however, negatively affect the performance of resistant individuals, when another stress factor appears because of the additional costs associated with mechanisms enabling them to counteract the effects of that stressor. Although many carabid beetles are carnivorous and actively seek prey, they can experience extended periods of starvation, especially in homogenized agricultural landscapes, dominated by large fields (Bilde and Toft 1998). Therefore, insects inhabiting such landscapes must cope not only with cyclic insecticide sprays but also with periodic starvation.

To answer the question about the cost of living under agricultural pressure and the possible costs of increased insecticide resistance observed in our earlier study (Sowa et al. 2022), biomarkers linked to metabolism (respiration rate) and physiology (acetylcholinesterase activity) were measured in the ground beetle *P. cupreus* originating from different habitat types located in two differently structured landscapes. We hypothesized that habitats with different intensities of agricultural pressure, represented here by canola coverage, influence beetles differently, depending on the structure of the surrounding large-scale agricultural landscape. We expected that high canola coverage, especially in the low complexity landscape dominated by large fields and scarcity of non-crop habitats, should result in increased metabolic rate and AChE activity due to the necessity to counteract the toxic effects of insecticides when no refuges (which may be provided by field boundaries) are readily available. We also tested the susceptibility of field-collected beetles to starvation, to check whether individuals from different habitat types differ in their maintenance costs. We conducted our study on *P. cupreus* for several reasons: (1) it is one of the most widespread carabid species in the agricultural landscapes studied, (2) it is an omnivorous species, considered an important service provider controlling pests and weeds (Kromp 1999, Frei et al. 2019), and (3) it has been proposed by the European Food Safety Authority as a sentinel species for pesticide risk assessment (EFSA 2021).

## Materials and methods

### Landscape and habitats selection

The detailed method of selecting the landscapes and habitat types within those landscapes has been described by Sowa et al. (2022). Briefly, within the farmland area located in the southwest Wielkopolska province in Poland, two differently structured landscapes, 12 × 16 km each, were selected: one dominated by large fields (4072 fields, 4442 km of field borders), hereafter ‘L’ or ‘low-complexity landscape’, and the second one with prevailing small-fields family farming (6490 fields, 6131 km of field borders), hereafter ‘S’ or ‘high-complexity landscape’ (Table 1).

**Table 1:** Characteristics of the two study landscapes, each 12 × 16 km, with the share of the main land cover types [%], the share of arable land in given field size classes [%], field border length [km] and the total number of arable fields (from Sowa et al. 2022)

		Low complexity landscape dominated by large fields (L)	High complexity landscape dominated by small fields (S)
Share of land cover units [%] in a landscape	Arable	73.2	76.9
	Herbaceous	8.8	8.2
	Woodland	11.2	8.7
	Build up	5.3	4.7
	Water bodies	0.8	0.6
	Other	0.7	0.9
Share of arable land [%] in given field size classes	<3 ha	23.5	41.7
	3–10 ha	25.7	40.1
	10–30 ha	21.7	11.5
	30–50 ha	16.8	4.8
	≥50 ha	12.3	1.9
Field borders [km]		4443	6131
Total number of arable fields		4072	6494

Within each landscape, three types of habitats, each in three replicates, were defined, based on the canola coverage (CC) expressed as the percentage of the total area within a 500 m radius around the midpoint where the beetle traps were located. In this work, we define “habitat” as the area experienced by the beetles (at least theoretically) during their lifetime. The average daily distance covered by beetles is 3–30 m and monthly 45–250 m (Thiele 1977), so the total area within a 500 m radius around the sampling midpoint (ca. 0.785 km^2^) refers to a population-level scale, whereas “landscape” type (ca. 192 km^2^) refers to a meta-population scale. In low-complexity landscape (L) the following habitat types were delineated: without canola (L0), with medium CC (28–33%, LM), and with large CC (80–98%, LL). In the high-complexity landscape (S) it was impossible to establish habitats with CC larger than 60% within a 500 m radius due to the lack of large canola fields and therefore the habitat types were distinguished as follows: without canola (S0), with small CC (10–14%, SS), and with medium CC (20–52%, SM). The midpoints (beetle traps) of study sites L0 and S0, serving as control sites, were located on meadows subjected to moderate agricultural practices with the exclusion of pesticide applications. The midpoints of the study sites were located in canola fields or meadows and were separated by at least 800 m to avoid catching beetles from one population at different study sites. The designated areas of 500 m radius did not overlap except in one case in a low-complexity landscape, where the edge of one of the L0 habitats overlapped slightly with one of the LM habitats. The whole area, covering both landscapes, is strongly dominated by conventional agriculture, with no organic-managed fields. Characteristics of the studied habitats are presented in Table 2 and Table S1. For each habitat we calculated the proportion of arable land [%] in given field size classes (<3 ha, 3–10 ha, 10–30 ha, ≥30 ha). As some fields were only partially within the 500 m radius buffer zones, fields with at least 20% of their area within the habitat were included in this analysis.

**Table 2:** Characteristics of study habitats, represented by individual areas within a 500 m radius around the midpoint where the beetle traps were located, selected in the two study landscapes, with the share of arable land in given field size classes [%] and the mean size of fields

Landscape type	Habitat type	Habitat No.	Share of arable land [%] in given field size classes				Mean size of fields [ha]
			<3 ha	3–10 ha	10–30 ha	≥30 ha	
High complexity landscape dominated by small fields (S)	S0	1	9.4	26.4	20.3	43.9	3.6
		2	51.1	48.9	0.0	0.0	
		3	58.8	41.2	0.0	0.0	
	SS	1	72.1	27.9	0.0	0.0	1.8
		2	45.2	54.8	0.0	0.0	
		3	49.4	50.6	0.0	0.0	
	SM	1	3.5	13.7	48.3	34.4	7.3
		2	29.4	21.5	0.0	49.0	
		3	32.5	67.5	0.0	0.0	
Low complexity landscape dominated by large fields (L)	L0	1	0.4	0.0	64.7	34.9	12.9
		2	6.6	4.5	41.1	47.7	
		3	1.2	0.0	24.7	74.1	
	LM	1	36.2	5.7	21.3	36.8	7.0
		2	6.0	16.0	48.7	29.3	
		3	0.0	33.5	66.5	0.0	
	LL	1	0.0	0.0	48.2	51.8	27.4
		2	0.4	3.5	32.3	63.8	
		3	0.0	0.0	26.6	73.4	

### Beetle collection

At the midpoint of each study site (18 study sites altogether), 64 Barber traps without any preservative were set up in a grid of 8 × 8 m (64 m^2^). The beetles were collected in 2018 during their peak activity in April–May. The traps were emptied every 2–3 days, the beetles were sorted in the field, and individuals of *P. cupreus* were placed in plastic containers (23 × 17 × 11 cm) with moist peat, transported to the laboratory and kept in a climatic chamber (20 ± 2 °C, relative humidity 70 ± 5%, day:night regime 16:8 h). To obtain sufficient numbers of beetles per habitat type, the beetles from three sites representing particular CC within a particular landscape type were pooled together. In the case of LL habitat, individuals were pooled from only two sites due to the very low number of individuals caught, some of which had to be allocated to other experiments. Beetles used for respiration rate measurement (and then for measurements of AChE activity) were caught two days before the measurements and were not fed. Those used to evaluate the response to starvation were fed ad libitum with artificial food made of frozen ground mealworms mixed with ground apples until the start of the experiment.

### Respiration rate, acetylcholinesterase activity and protein content in the beetles

In total, the respiration rate of 180 adult males was measured: S0–47, SS–43, SM–12, L0–47, LM–20, and LL–11. After being weighed to the nearest 0.0001 g (Radwag WPA 180/K, Poland), the beetles were placed individually into 50 ml flasks connected to a 30-channel Micro-Oxymax respirometer (Columbus Instruments, USA). A punctured Eppendorf tube filled with distilled water was placed in each flask to prevent beetle desiccation. The animals were not supplied with food during the measurements and all measurements were performed at 20 ± 1 °C and day:night regime 16:8 h. The respiration rate was measured at 4 h intervals for 28 h as oxygen consumption per hour per beetle and then recalculated per gram body mass for data analysis (μl O_2_ g^−1^ h^−1^). Before analysing respiration rates, the first measurement point (the first 4 h interval) for each individual was excluded from the data because changing the environment and handling stress might cause temporarily abnormal activity and respiration rates.

Because of the limited number of respirometer channels, the measurements were taken in six series. As it was not always possible to ascertain enough individuals from all habitat types from both landscapes, in the first series only beetles from the S landscape were used, in the second – from the L landscape, and then alternately until the sixth series. In the last series, specimens from both landscapes were present fifty-fifty. After the measurements, the beetles were weighed again, and put individually into 2 ml plastic tubes (Sarstedt AG & Co. KG, Germany), frozen in liquid nitrogen and stored at −80 °C for acetylcholinesterase activity measurements. The body mass after respiration rate measurements was used to normalize the total protein content in the beetle by dividing it by body mass (mg g^−1^).

Acetylcholinesterase activity and protein contents were measured in 175 out of 180 beetles for which respiration rate was measured because 5 samples were lost during the homogenization. Into all tubes with frozen beetles, 1 ml of ice-cold 0.05 M phosphate buffer (pH 7.4) and five 2.8 mm ceramic beads (Omni International, USA) were added. The samples were homogenized for 1 min using a mechanical homogenizer (Bead Ruptor Elite, Omni International, USA). The homogenates were centrifuged at 4 °C for 15 min at 15,000 g (MPW-350R, MPW MED Instruments, Poland). The supernatant from each sample was transferred to a new 2 ml Eppendorf tube (Sarstedt AG & Co. KG, Germany) and used for AChE activity and protein content analyses. Acetylcholinesterase activity was measured according to the modified method by Ellman et al. (1961) on 96-well plates (Sarstedt, USA), using the Synergy HTX multi-mode reader spectrometer (BioTek Instruments, Inc., USA). The reaction medium was made for each sample by mixing 10 µL of supernatant with 175 µL of phosphate buffer (0.05 M, pH = 7.4) and 10 µL 0.01 M DTNB (5,5′-dithiobis(2-nitro-benzoic acid); Sigma-Aldrich, Germany) solution prepared in the 0.1 M Tris-HCl (pH 8.0, Sigma-Aldrich, Germany). The reaction was initiated by adding to each well 5 µL of solution of 0.1 M acetylthiocholine iodide ((2-mercaptoethyl) trimethylammonium iodide acetate; Sigma-Aldrich, Germany) prepared in double distilled water as a substrate. After adding the substrate, the plate was shaken for 6 s, then incubated for 150 s at 22 °C and the absorbance at 405 nm was then measured in 42 s intervals for 260 s. The reaction medium with phosphate buffer (0.05 M, pH 7.4) was used as a negative control. AChE activity was measured in four replicates for each sample and the outermost measurement was excluded for the calculation of the mean AChE activity. This was done by calculating the average at each time interval. By using the calculated average, an absolute value was calculated for each measurement and then a maximum one for each time point was extracted. Then, by applying the criterion of whether the maximum value is equal to the absolute value, a 0–1 matrix was created to determine which values are closest to the mean, allowing the identification of a measurement that should be discarded. The mean of the remaining three readings per sample, expressed as µmoles acetylcholine hydrolysed per min per mg protein (µmoles min^−1^ mg^−1^ protein), were used in the statistical analysis of AChE activity.

Protein content in the same supernatant as used for AChE analysis was assessed using Bradford’s reagent (Sigma, USA) at a 1:50 supernatant:reagent ratio. The absorbance was measured at 600 nm after incubation of the plate for 5 min at 25 °C, using bovine serum albumin (BSA standard, Sigma, USA) as a standard.

### Response of beetles to starvation

Due to availability, in this experiment, beetles (without distinguishing between the sexes) collected in four habitat types were used: S0 (*n* = 40), SS (*n* = 40), L0 (*n* = 40) and LM (*n* = 30), i.e., 150 individuals in total. The beetles were placed individually in plastic Petri dishes (diameter 35 mm, FL Medical, Italy) and kept in a climatic chamber (20 ± 2 °C; relative humidity 70 ± 5%, day:night regime 16:8 h). Every second day each individual was supplied with a 30 µl droplet of tap water. Survival was recorded daily until the last beetle died. The dead beetles were frozen in 2 ml Eppendorf tubes at −20 °C until they were dried at 105 °C for 24 h and weighed to the nearest 0.0001 g.

### Statistical analysis

Respiration rate, acetylcholinesterase activity, protein content and dry mass of beetles from different habitat types were compared using GLM mixed model, with habitat type as the main experimental factor and site as the random factor nested within habitat type. For respiration rate analysis, beetle body mass was added to the model as the quantitative factor to account for the allometry. The distribution of residuals was checked for normality with Shapiro-Wilk’s W test. When this condition was not met, statistical analyses were performed on log-transformed data (acetylcholinesterase activity and respiration rate). Means were separated with the Tukey post hoc test.

In the starvation experiment, the exact collection site of individual beetles was not recorded so we could not evaluate the possible effect of the random factor. Hence, in this case all statistical analyses were done exclusively at the level of habitat type. Survival curves of beetles originating from different habitat types were compared using survival analysis with the Logrank test. The Logrank test was chosen because it performs well when the underlying distributions have constant hazard ratios (Lee et al. 1975, Tarone and Ware 1977). In the first step, data for all six habitats were analysed together for the effect of habitat type. As statistically significant differences were found (*p* < 0.008), this was followed by pairwise comparisons of each habitat type against one another. All statistical analyses were performed using Statgraphics Centurion 19 (Statgraphics Technologies, Inc., USA) at a 95% confidence level.

## Results

**Fig. 1:**
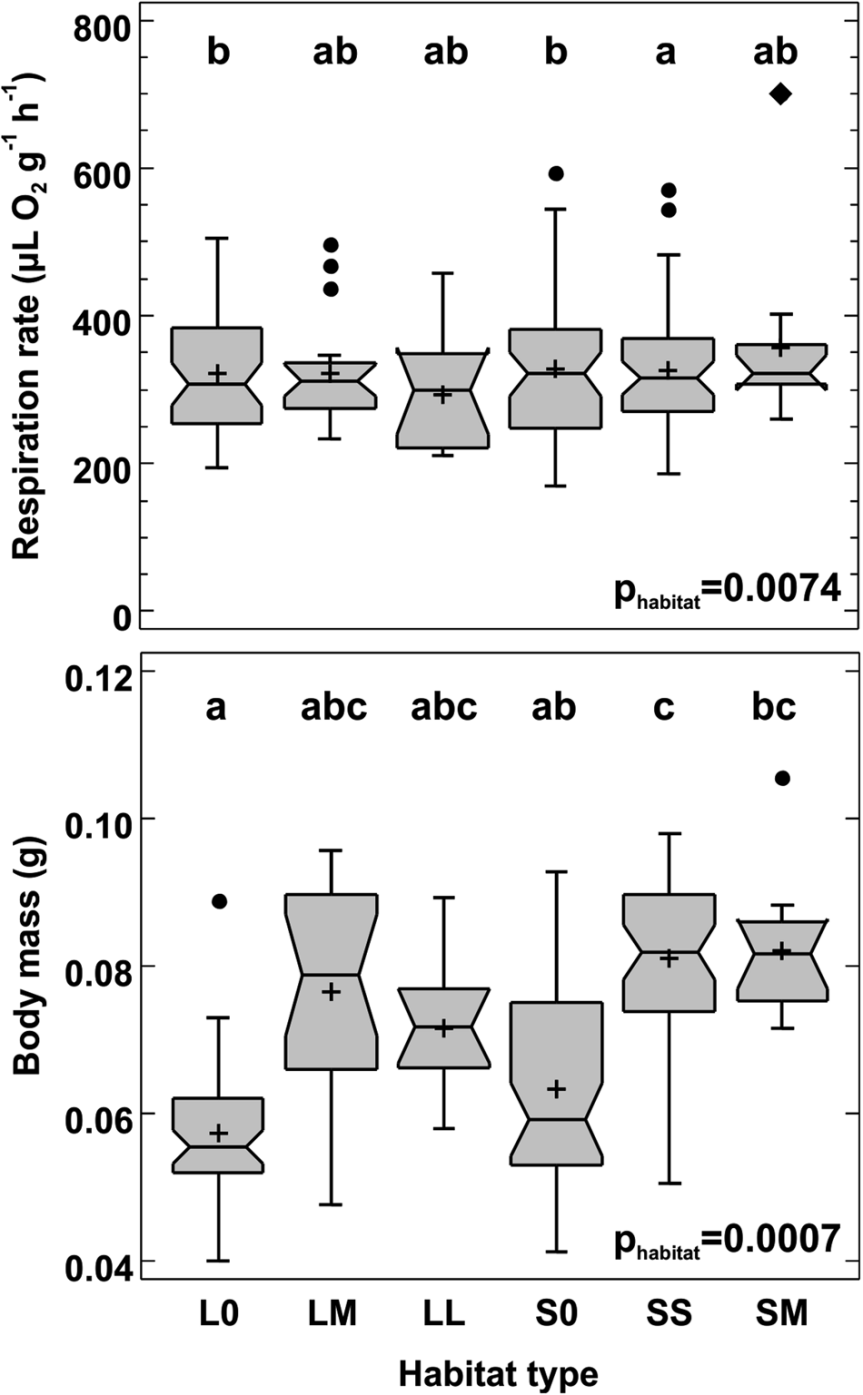
Respiration rate (top) and body mass (bottom) of *Poecilus cupreus* from different habitat types. In the *x*-axis labels the first character stands for landscape type (L – large-fields landscape, S – small-fields landscape), the second for canola coverage (0 – none, S – small (10–14%), M – medium (20–52%), L – large (80–98%)). Number of individuals used from each habitat: S0–47, SS–43, SM–12, L0–47, LM–20 and LL–11. The graphs show median (shorter horizontal line), average (plus sign), lower and upper quartile (longer horizontal lines), minimum and maximum values (whiskers) except for any outliers >1.5 interquartile range (circles) or far outliers >3 interquartile range (diamond); the notch indicates an approximate 95% confidence interval for the median; p – significance level for the effect of habitat type on the mean log(Respiration) and mean body mass (GLMM). Beetles from the habitats marked with the same letter above box-and-whisker plots do not differ significantly at *p* ≤ 0.05 (Tukey test)

All individuals survived the respiration rate measurements. The respiration rate corrected for body mass differed significantly between habitat types (F_5,162_ = 4.20, *p* = 0.007), with the non-significant effect of the random factor (site F_11,162_ = 0.87, *p* = 0.57) and significant effect of body mass (F_1,162_ = 33.36, *p* < 0.0001); the *p* value for the full model was 0.0003. The LM, LL and SS beetles had the lowest respiration rate, and S0 and L0 the highest, but statistically significant differences were found only between SS and S0 and between SS and L0 habitats (see Fig. 1 for the Tuckey post hoc test). Body mass also differed between the habitat types (F_5,163_ = 9.10, *p* = 0.0007), controlled for the significant random site effect (F_11,163_ = 3.38, *p* = 0.0003); the model including both factors was significant at *p* < 0.0001. Beetles from canola-free habitats S0 and L0 had the lowest body mass, those from SS and SM habitats the highest (Fig. 1).

**Fig. 2:**
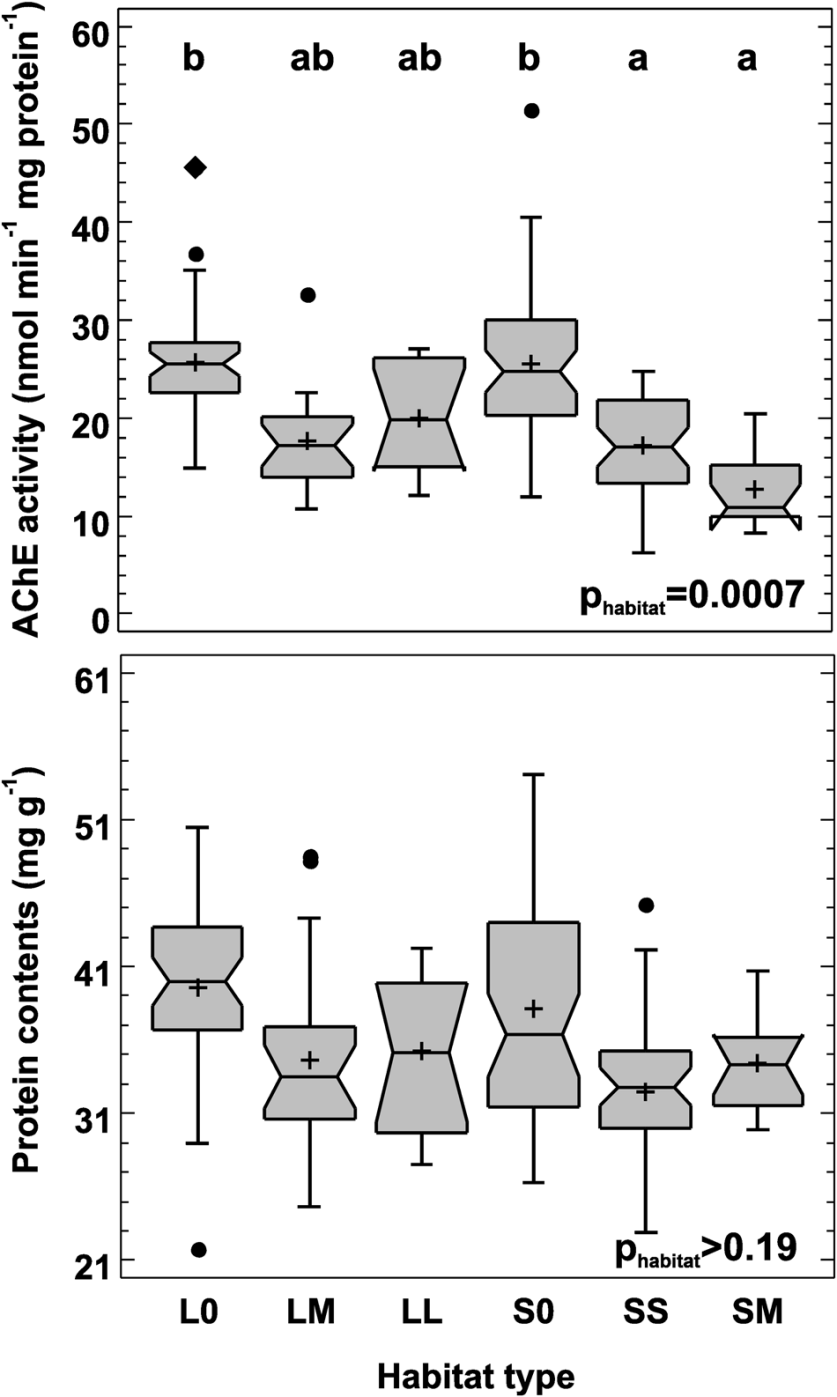
Acetylcholinesterase activity (AChE; top) and protein content per body mass (bottom) in *Poecilus cupreus* from different habitat types. In the x-axis labels the first character stands for landscape type (L – large-fields landscape, S – small-fields landscape), the second for canola coverage (0 – none, S – small (10–14%), M – medium (20–52%), L – large (80–98%)). Number of individuals used from each habitat: S0 – 47, SS – 42, SM – 12, L0 – 43, LM – 20 and LL – 11. The graphs show median (shorter horizontal line), average (plus sign), lower and upper quartile (longer horizontal lines), minimum and maximum values (whiskers) except for any outliers >1.5 interquartile range (circles) or far outliers >3 interquartile range (diamond); the notch indicates an approximate 95% confidence interval for the median; p – the significance level for the difference in mean log(AChE) and protein levels between habitat types (GLMM). The means of groups marked with the same letter above box-and-whisker plots do not differ significantly at *p* ≤ 0.05 (Tukey test)

The GLMM showed a significant habitat effect (F_5,158_ = 8.80, *p* = 0.0007) on mean acetylcholinesterase activity (Fig. 2), controlled for the random site effect (F_11,158_ = 2.41, *p* = 0.008); the model including both factors was significant at *p* < 0.0001. Beetles from both habitats without canola (S0 and L0) had the highest AChE activity and differed significantly from SS and SM habitats. The lowest AChE activity was found in SM beetles, but it did not differ significantly from SS, LM and LL beetles (Fig. 2).

Although the GLMM for protein content was also significant (F_16,158_ = 5.35, *p* < 0.0001), this was solely due to the random site effect (F_11,158_ = 3.63, *p* = 0.0001), with the non-significant habitat type effect (F_5,158_ = 1.74, *p* > 0.19). Nevertheless, median protein contents were the highest in L0 and S0 beetles (Fig. 2).

**Fig. 3:**
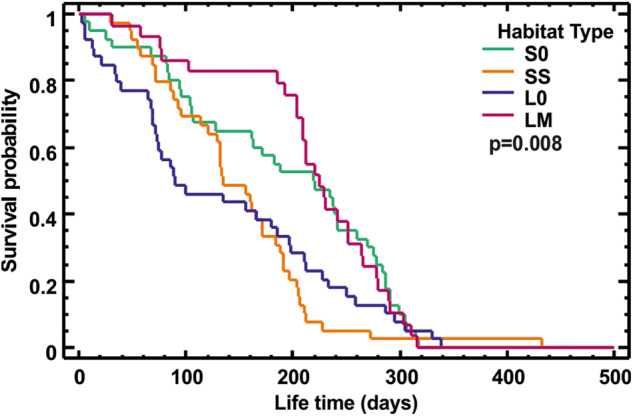
Survival curves of starving *Poecilus cupreus* beetles from different habitat types. The first character in the habitat type legend stands for landscape type: S – small-fields landscape, L – large-fields landscape; the second character stands for canola coverage: 0 – none, S – small (10–14%), M – medium (20–52%). The number of individuals used from each habitat: S0–40, SS–40, L0–40, LM–30; p – significance level for the difference in the survival curves between habitat types

**Fig. 4:**
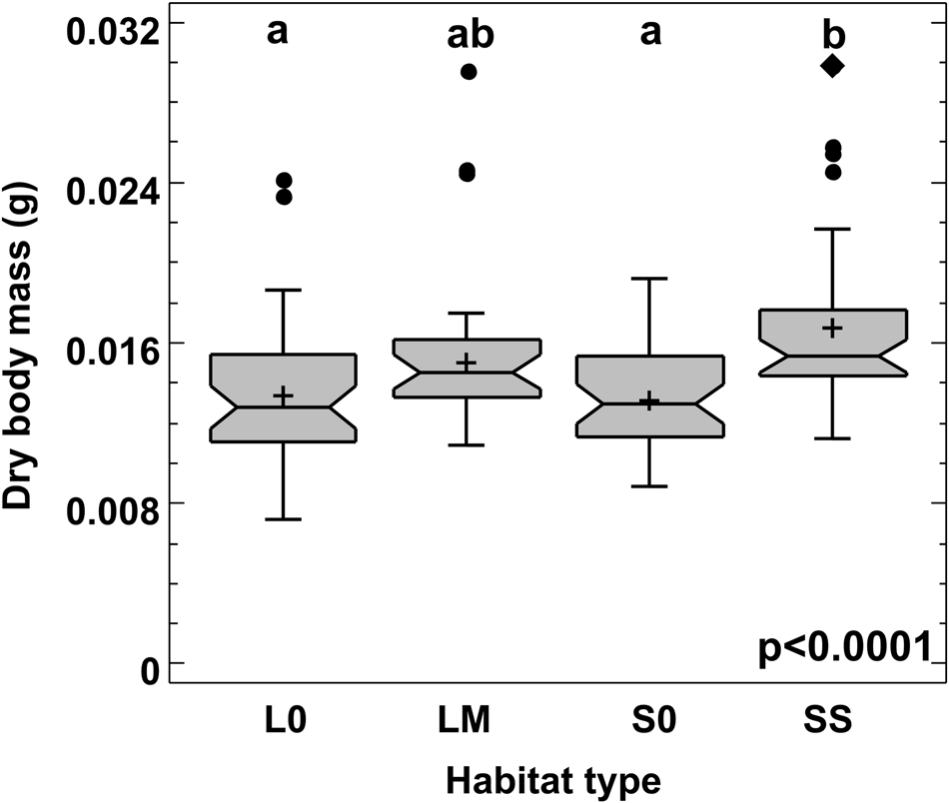
Dry body mass of *Poecilus cupreus* from the four different habitat types. In the *x*-axis labels the first character stands for landscape type (L – large-fields landscape, S – small-fields landscape), the second for canola coverage (0 – none, S – small (10–14%), M – medium (20–52%)). Number of individuals used from each habitat: S0–40, SS–40, L0–40, LM–30. The graphs show median (shorter horizontal line), average (plus sign), lower and upper quartile (longer horizontal lines), minimum and maximum values (whiskers) except for any outliers >1.5 interquartile range (circles) or far outliers >3 interquartile range (diamond); the notch indicates an approximate 95% confidence interval for the median; p – significance level for the difference in mean dry body mass between habitat types (GLM). The means of groups marked with the same letter above box-and-whisker plots do not differ significantly at *p* ≤ 0.05 (Tukey test)

Both populations from canola-free habitats, representing control habitats with no insecticide treatments, showed similar survival rates when starving (*p* = 0.11, Fig. S1), meaning that the background mortality was the same in both landscapes. When populations from all habitat types were compared together, the Logrank test showed, however, a significant (*p* = 0.008) habitat effect on survival rates (Fig. 3). Pairwise comparisons of the habitats with one another revealed that beetles from LM habitat survived significantly better than those from other habitat types except S0, while those from SS survived better than their corresponding control (S0) (Fig. 3, Table 3). The median lifetime (LT_50_) for beetles from the LM habitat was almost three times higher than for beetles from the L0 and ca. two times higher than for those from the SS (Table 3). In the starvation experiment, the dry body mass of beetles from SS habitat was significantly higher than of beetles from each of the control habitats (S0 & L0) (*p* < 0.0001, Fig. 4).

**Table 3:** Lethal times (LT25, LT50 & LT90, days) of starving *Poecilus cupreus* from four habitat types

Site	*N*	LT25	LT50	LT90	Logrank test
S0	40	278	219	30	ac
SS	40	191	134	54	b
L0	40	212	89	12	ab
LM	30	266	224	75	c

The sites are named as follows: the first character stands for landscape type (S – small-fields landscape, L – large-fields landscape), the second for canola coverage (0 – none, S – small (10–14%), M – medium (20–52%)). Groups with the same lowercase letter do not differ significantly at *p* ≤ 0.05 in survival (pairwise comparison of survival curves, Logrank test); N – initial number of individuals

## Discussion

The overall goal of this study was to assess the costs of living in differently structured agricultural landscapes. The degree of risk posed by pesticide sprays depends not only on local management practices but also on the context of agriculture and land use at the landscape level (Seibold et al. 2019). Therefore, the strongest changes in the beetle metabolism and physiology were expected in individuals from LL habitats, where the presence of large canola monocultures and fewer boundaries (bulks) between fields creates a situation in which beetles deprived of refuges are chronically exposed to pesticides.

Our previous study has shown that agricultural intensification and the associated use of pesticides lead to elevated resistance in *P. cupreus* to the selected insecticide. We observed this phenomenon in individuals from habitats with a medium to high share of canola in the low- complexity landscape (Sowa et al. 2022). Developing such a resistance mechanism must be costly (Sibly and Calow 1989), so to address the question about those costs, biomarkers linked to metabolism and physiology were measured in the beetles collected from different habitat types located in two contrasting landscapes. Neither energy expenditure, measured as respiration rate, nor AChE activity was affected in a way that would explain the better tolerance towards insecticide in beetles from canola habitats in the large-fields landscape observed previously. However, beetles from the low-complexity landscape showed better survival during starvation than those from the high-complexity landscape, indicating that individuals from the landscape of lower complexity must have adapted in some way to the adverse conditions through the increased resistance and better resource management.

In contrast to our expectations, the mean respiration rate was the highest in beetles from the control habitats, S0 and L0 (Fig. 1). As the respiration rate measurements covered 24 h, they included both the resting and active metabolic rates. One may speculate, thus, that S0 and L0 beetles were more active, resulting in a slightly higher respiration rate on average, although significantly different from SS site only.

Whole body respiratory metabolism is one of the core components of an individual’s energy budget. Because it reflects overall maintenance costs, it should be sensitive to any internal and external stressing factors (Manga 1972, Todd 1997). Hence, changes in respiration rate can indirectly measure additional maintenance costs due to exposure to such factors. The depletion of energy reserves or increase in metabolic rates is, thus, expected to be associated with the toxicity of insecticides. Kivimägi et al. (2013) in their study on another carabid species, *Platynus assimilis*, demonstrated that beetles subjected to sub-lethal doses of alpha-cypermethrin had significantly increased resting metabolic rate. The opposite reaction was found, however, in the moth *Anticarsia gemmatalis* – a soybean pest, which showed a decrease in the respiration rate after exposure to chlorpyrifos (Plata-Rueda et al. 2020). However, it is important to note that in both studies cited above, the respiration rate was measured immediately after the exposure to insecticides. In contrast, in our study, the beetles were collected from the field and were not treated with insecticides in the laboratory before respiration rate measurements.

In their seminal work, Sibly and Calow (1989) presented the idea of energy costs connected with the reallocation of resources to processes favouring tolerance to stress (e.g., defence against pesticides) or repairing damages. Briefly, exposure to a toxicant depletes the energy resources available for maintenance, growth and/or reproduction by diverting part of the energy to detoxification and/or repair. Surprisingly, the beetles from habitats without canola not only had the highest mean respiration rate (although significantly different from SS habitat only) but were also the lightest among all populations. The difference in mass between individuals from different habitat types may be due to *Poecilus cupreus* being a species that prefers arable fields to other land cover types. Canola fields, despite being sprayed, potentially provide greater seed availability and prey numbers that are more readily available. Interspecific competition and differences in microclimate between meadows and canola fields could be another factor influencing the difference in beetles’ mass.

The fact that respiration rates of beetles from canola habitats were either similar or even lower than in S0 and L0 beetles suggests that energetic costs of insecticide detoxification are too low to be detected with the method used or that they were compensated by some shifts in the energy budget, e.g., lower locomotor activity. Depending on the situation (e.g., stress caused by organic vs. mineral or biodegradable vs. persistent compound), organisms can deploy a range of different detoxification techniques. Additionally, as shown by Sowa et al. (2022), beetles from the low-complexity landscape exhibited increased resistance to selected insecticides. We can assume, thus, that the multi-generation evolution of detoxification can develop in a way that helps in keeping the energy budget on a fairly stable level. If this was the case, changes in detoxification processes would not necessarily manifest in a decrease or increase in respiration rate but, for example, in decreased reproduction.

One of the best biomarkers of the non-lethal effects of insecticides impairing the function of the nervous system is a change in acetylcholinesterase activity (Boily et al. 2013, Pundir and Chauhan 2012). On the one hand, AChE inhibition could be expected in the beetles collected from the field shortly after exposure to insecticides, especially in the case of exposure to organophosphorus and carbamate insecticides. On the other hand, multigenerational exposure of populations inhabiting agricultural areas could lead to inherited changes in physiology, manifested as increased AChE activity to cope with repeated insecticide exposures (Mohamadi et al. 2010). We expected that the latter effect should be visible particularly in beetle populations from large canola fields in the L landscape, where fewer or no refuges are available to evade the selective pressure of insecticides. In contrast to this expectation, AChE activity in beetles from the LL and LM habitats did not differ significantly from both populations without canola (L0 and S0). However, AChE activity was significantly lower in the SS and SM populations compared to controls (L0 and S0). As *P. cupreus* from all canola habitats did not differ from one another in terms of AChE activity and tended towards lower enzyme activity than in S0 and L0 beetles, this may suggest that beetles from all canola habitats either have indeed developed elevated resistance to insecticides through some shifts in energy allocation or are permanently affected by insecticides suppressing AChE activity. Although our main gradient was defined by canola coverage, we cannot also exclude that some of the observed differences between controls (S0, L0) and the remaining habitats stem from other characteristics than just the absence of canola.

Our results showed that starving beetles from the LM habitat lived longer than those from the L0 and SS habitats. This is an intriguing finding since the LM beetles came from habitats with relatively high canola coverage located in the low-complexity L landscape with a scarcity of uncultivated elements, such as grassy field margins, playing a significant role in food acquisition by ground beetles (Zangger 1994, Haschek et al. 2012). A wide range of prey that can be found in uncultivated areas should be favourable, offering a better, more diverse food supply, allowing not only for higher beetle densities (Holland 2002) but also assuring lower pressure on metabolic changes and disturbances in energy reserves due to chronic exposure to insecticides. In high-quality environments, like, presumably, meadows in L0 and S0 habitats, there is no evolutionary pressure favouring survival during prolonged periods of food shortages. A similar mechanism may also apply to the beetles from the SS habitat due to the availability of non-crop landscape elements (field boundaries) within the range of short-term dispersion. As shown by Juliano (1986), individuals of *Brachinus* species in case of a prolonged period of starvation or water stress either disperse or die. Similar behaviour was found in populations of ladybirds *Coleomegilla maculata* (Nault and Kennedy 2000) which migrate from corn to potato fields. The dispersion of insects in both these studies was not due to some species-specificity, but the uneven spatial and temporal distribution of prey or other resources and changes in plant cover compel the beetles to continuously search for alternative food sources for year-round survival. Therefore, a heterogeneous landscape with a large number of small fields, and thus greater length of boundaries between fields, should significantly compensate for the most unfavourable conditions (e.g., starvation).

Living in conditions that are highly disturbed adapts organisms to the more rational use of their resources. As shown by Lövei et al. (1985), to survive prolonged periods of starvation, larvae of *P. cupreus* hold the food in their alimentary canal to use the consumed food more efficiently. Adaptations to minimize energy demand, as in the case of hibernation (Pond 1981), become an excellent mechanism for beetles inhabiting highly simplified landscapes. Such energy management has, however, its consequences. Selection for resistance to starvation and/or desiccation can alter the patterns of early fecundity and change larvae viability and development time (Knapp and Uhnavá 2014). Van Dijk (1986) showed that in the related species, *Poecilus versicolor*, females lay eggs only after feeding. This indicates the presence of a trade-off between maintenance costs and reproduction. The better survival of starving beetles from the LM habitat could relate to higher body weight, as it is known that increased lipid content indeed increases the resistance to starvation. However, the dry body mass of beetles from the LM (and S0) habitats did not differ from the other two groups studied, suggesting that this is not the determining factor in this case. In general, compared to other carabid species, *P. cupreus* is one of the best-performing species, surviving for twice as long as *Anchonemus dorsalis* (Knapp 2016) and three to four times longer than *Merizodus soledadinus* and *Calosoma sayi* (Young 2008, Laparie et al. 2012) under starvation. Selection for increased resistance to starvation has been already proven in adult *Drosophila melanogaster* (Rose et al. 1992), demonstrating that such selection also increased longevity which is on par with our results.

## Conclusion

We observed a significant decrease in AChE activity in populations of beetles from habitats with canola (SS and SM) in high-complexity landscape. This suggests that beetles may have undergone physiological and/or biochemical changes. They could be permanently affected by insecticides, resulting in suppressed AChE activity. Importantly, our results showed no discernible differences in AChE activity between populations from canola fields and different landscape types, indicating that landscape structure alone is not sufficient to counteract pesticide pressure. Contrary to our expectations, we did not find higher respiration rates in *P. cupreus* populations inhabiting low-complexity landscape with canola habitats. Together, these two results did not provide a clear explanation for the increased insecticide tolerance to insecticides reported in our earlier study (Sowa et al. 2022), nor did they indicate the potential costs associated with this tolerance. We found no deterioration in the performance of beetles from the low-complexity landscape under starvation conditions. Surprisingly, the beetles from the LM habitat performed as well as those from S0 and even better than those from L0 and SS habitats, suggesting their selection for increased resistance to starvation. In general, this and our previous study (Sowa et al. 2022) suggest that the beetles may have undergone physiological and/or biochemical adaptations to increase their resistance to insecticides, although the exact mechanisms and associated costs remain unclear.

### Supplementary information

Supplementary Information
