# Supplementary material for: Effects of agricultural landscape structure and canola coverage on biochemical and physiological traits of the ground beetle Poecilus cupreus
Source: Ecotoxicology. 2023 Sep 27;32(9):1141–51. doi: 10.1007/s10646-023-02701-3 (PMC10684619; doi:10.1007/s10646-023-02701-3)
Supplement: Supplementary file 1 — Supplementary Information [file 10646_2023_2701_MOESM1_ESM.docx]

**SUPPLEMENTARY INFORMATION**

Effects of agricultural landscape structure and canola coverage on biochemical and physiological traits of the ground beetle *Poecilus cupreus*

Grzegorz Sowa^1^, Agnieszka J. Bednarska^2^, Ryszard Laskowski^1^

^1^ Institute of Environmental Sciences, Jagiellonian University, Gronostajowa 7, 30-387 Kraków, Poland

^2^ Institute of Nature Conservation, Polish Academy of Sciences, A. Mickiewicza 33, 31-120 Kraków, Poland

Corresponding author: Grzegorz Sowa

e-mail: [grzegorz.sylwester.sowa@gmail.com](mailto:grzegorz.sylwester.sowa@gmail.com)


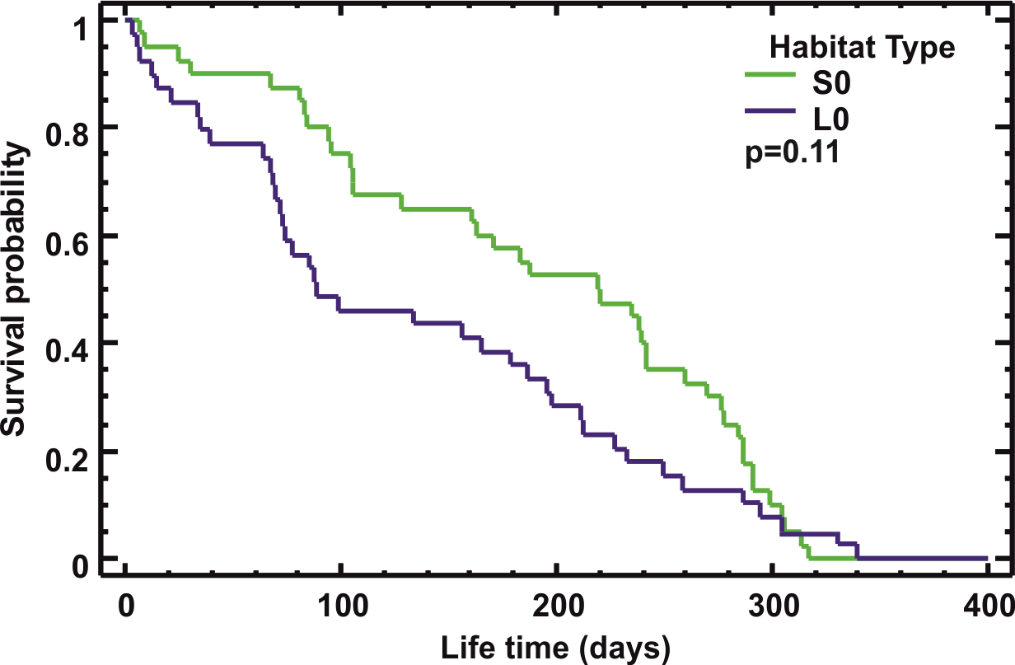


**Figure S1.** Survival curves of starving *Poecilus cupreus* beetles from control habitats (0 in the legend stands for no canola within a 500 m radius) in two landscapes: S – small fields landscape, L – large fields landscape. Number of individuals used from each habitat – 40. The p – significance level for the difference in the survival curves between the habitat types.

**Table S1.** Characteristics of habitats, each of the area within a 500 m radius around the midpoint where the beetle traps were located, selected in the two study landscapes, with the share of canola and the main land cover types [%].

| **Landscape type** | **Habitat type** | **Habitat No.** | **Canola coverage [%]** | **Share of land cover units [%] in a habitat** | | | | | | |
| --- | --- | --- | --- | --- | --- | --- | --- | --- | --- | --- |
|  |  |  |  | Arable | Herbaceous | Woodland | Build up | Water bodies | Other |  |
| **High configuration landscape**  **dominated by small fields (S)** | S0 | 1 | 0.0 | 73.3 | 22.5 | 0.9 | 0.5 | 1.5 | 1.3 |  |
|  |  | 2 | 0.0 | 57.7 | 24.2 | 4.7 | 11.9 | 0.6 | 1.0 |  |
|  |  | 3 | 0.0 | 53.7 | 33.2 | 1.1 | 10.2 | 1.1 | 0.7 |  |
|  | SS | 1 | 14.5 | 82.4 | 4.0 | 3.8 | 8.6 | 0.6 | 0.7 |  |
|  |  | 2 | 13.0 | 90.9 | 1.0 | 0.9 | 6.0 | 0.0 | 1.2 |  |
|  |  | 3 | 10.8 | 94.9 | 1.9 | 0.5 | 1.6 | 0.5 | 0.6 |  |
|  | SM | 1 | 52.0 | 98.4 | 0.9 | 0.4 | 0.1 | 0.1 | 0.1 |  |
|  |  | 2 | 28.4 | 91.9 | 3.2 | 0.8 | 2.4 | 0.9 | 0.7 |  |
|  |  | 3 | 20.4 | 64.0 | 24.6 | 0.9 | 7.0 | 1.5 | 2.0 |  |
| **Low configuration landscape**  **dominated by large fields (L)** | L0 | 1 | 0.0 | 62.1 | 25.1 | 2.7 | 2.6 | 7.3 | 0.3 |  |
|  |  | 2 | 0.0 | 45.6 | 18.8 | 5.8 | 28.2 | 0.9 | 0.6 |  |
|  |  | 3 | 0.0 | 65.6 | 12.8 | 4.6 | 3.9 | 12.6 | 0.6 |  |
|  | LM | 1 | 36.7 | 96.1 | 1.7 | 0.6 | 1.1 | 0.1 | 0.4 |  |
|  |  | 2 | 26.8 | 95.1 | 1.1 | 2.0 | 1.4 | 0.0 | 0.4 |  |
|  |  | 3 | 32.8 | 63.7 | 30.3 | 0.8 | 2.8 | 2.1 | 0.3 |  |
|  | LL | 1 | 98.5 | 97.4 | 1.4 | 0.4 | 0.6 | 0.0 | 0.2 |  |
|  |  | 2 | 80.9 | 92.2 | 0.4 | 4.9 | 2.0 | 0.3 | 0.1 |  |
|  |  | 3 | 87.4 | 90.1 | 5.6 | 1.8 | 1.5 | 0.2 | 0.9 |  |
